# Factors Associated with Apgar Score among Newborns Delivered by Cesarean Sections at Gandhi Memorial Hospital, Addis Ababa

**DOI:** 10.1155/2020/5986269

**Published:** 2020-01-06

**Authors:** Mohammed Suleiman Obsa, Getahun Molla Shanka, Misrak Woldayohannes Menchamo, Robera Olana Fite, Meron Abrar Awol

**Affiliations:** ^1^Department of Anesthesia, Wolaita Sodo University, Wolaita, Ethiopia; ^2^School of Medicine, Wolaita Sodo University, Wolaita, Ethiopia; ^3^Addis Ababa University School of Anesthesia, Addis Ababa, Ethiopia; ^4^Department of Nursing, Wolaita Sodo University, Wolaita, Ethiopia

## Abstract

**Background:**

Newborns can be assessed clinically using the Apgar score test to quickly and summarily assess the health of newborn physical condition immediately after delivery and to determine any immediate need for extra medical or emergency care. This study is aimed at assessing factors associated with Apgar score among newborns delivered by cesarean sections and factors associated with Apgar score.

**Method:**

Institutional-based cohort study design was conducted. All eligible study participants were included. Training was given for data collectors and supervisors. Regular supervision and follow-up was made. Data was entered into Epi Info version 7 computer software by investigators and was transported to SPSS version 20 computer program for analysis. Bivariate and multivariate analysis was used to identify factors associated with Apgar score.

**Result:**

A total 354 newborn babies were included into the study. Majority of baby had low Apgar score at one minute and high Apgar score at five minutes. About 30.2% of newborn baby had Apgar score below seven minutes. On the other hand, about 12.8% of all newborns had low Apgar score at five minutes. It had been found that those neonates who were born when skin incision to delivery time is greater than three minutes were about fourfolds more likely to have low Apgar score than those who were born when skin incision to delivery time is less than three minutes (AOR 3.645) (95% CI (0.116-26.421)).

**Conclusion:**

Newborn babies have a low Apgar score at one minute as compared to five minutes. But low Apgar score at five minutes has long-term sequel. Therefore, it is very important to reduce factors associated with low Apgar score at both minutes.

## 1. Introduction

Newborns delivered by cesarian section (CS) can be assessed clinically using the Apgar score which was devised in 1952 by Dr. Virginia Apgar to evaluate the health of newborn and assess the effects of obstetric anesthesia on newborns at birth [1]. The test is simple and repeatable method to quickly and summarily assess the health of newborn physical condition immediately after delivery and to determine any immediate need for extra emergency care [2, 3].

Five factors are used to evaluate the baby's condition and each factor is scored on a scale of 0 to 2, with 2 being the best score for each: the scoring system is an accepted tool for assessing the vitality of newborn infants. The score is based on measures of heart rate, respiratory effort, skin color, muscle tone, and reflex irritability [4].

Scores obtainable are between 0 and 10, with 10 is the highest possible score. A total score of 7-10 is considered “normal,” and a lower Apgar score indicates depressed vitality [5]. However, several possible causes of low Apgar scores exist, such as perinatal asphyxia and congenital infections [6–9].

Apgar score is usually done to the baby twice: once at one minute after birth and again at 5 minutes after birth. Rarely, if there are concerns about the baby condition and the first two scores are low, less than 7, the scoring is also performed at 10, 15, and 20 minutes after delivery [10, 11].

The 1 min Apgar score may signal the need for immediate resuscitation, and the Apgar score measured 5 min after birth is a predictor of neonatal mortality and several neurological outcomes [12–14].

## 2. Method and Materials

### 2.1. Study Setting

Cohort study design was conducted from January to March 2016 at Gandhi.

### 2.2. Source Population

This section discusses all newborn babies of mothers who gave birth by cesarian section at Gandhi Memorial Hospital, Addis Ababa.

### 2.3. Study Population

This section discusses selected newborn baby of mothers who gave birth by cesarian section from January to March at Gandhi Memorial Hospital, Addis Ababa.

### 2.4. Exclusion Criteria

This section discusses acute fetal distress, intrauterine fetal death, pregnancies with bleeding, and a mother who refused to take part in the study.

### 2.5. Data Collection Tools and Procedure

Data was collected using pretested structured questionnaires. Apgar score was done as per the protocol prescribed by the neonatal Advanced Life Support advocated by the American Pediatric Association. At delivery, for evaluation of neonate, Apgar scores were assigned at one and five minutes. It was based on the appearance (color), pulse rate, grimace (reflexes), muscle tone (activity), and respiratory effort of neonate each carrying a score from 0 to 2 (Table 1).

Apgar score scaling based on neonatal advanced life support is advocated by the American Pediatric Association (APA) [10].

### 2.6. Data Quality Assurance

The questionnaire was prepared in English first and translated to the local language, Amharic, and again back to translation to English was made to ensure that the consistency of the question. Pretest was done on 5% of the sample size at Zewditu Memorial Hospital. Data collectors and supervisors were trained on each items included in the study tools. Double entry was made on 10% of sample size.

### 2.7. Data Analyzing and Processing

The data was entered into Epi Info version 7 and transported to SPSS version 20 computer program for analysis. Bivariate and multivariate analysis was used to see the effect of independent variable over Apgar score. Variables which were significant on bivariate analysis at *p* value less than 0.2 were taken to multivariate analysis. In multivariate analysis *p* value of less than 0.05 was used as a cut of point for the presence of association. Strength of association was measured by 95% confidence interval and odd ratio.

### 2.8. Ethical Consideration

Ethical clearance and approval was obtained from ethical review committee, Anesthesia Department, Addis Ababa University. Permission to conduct was obtained from Gandhi Memorial Hospital. Informed verbal consent was secured from study participants. The obtained data was only used for study purpose. Confidentiality and anonymity were ensured.

### 2.9. Operational Definition

#### 2.9.1. Cesarian Section

An intentional incision made on an intact uterus to delivery fetus.

#### 2.9.2. Newborn

It refers specifically to the infant in the first minutes to hours after birth.

#### 2.9.3. Neonate

It generally defined as an infant during the first 28 days of life. Infancy includes the neonatal period and extends through 12 months of age.

#### 2.9.4. High Apgar Score

This section discusses Apgar scores of 7 or above.

#### 2.9.5. Low Apgar Score

This section discusses Apgar scores below 7.

## 3. Result

### 3.1. Sociodemographic and Personal Characteristics

A total 344 pregnant mothers gave birth to 354 newborns at Gandhi Memorial Hospital of which 37.20% were primigravida and 48.84% were multigravida. Pertaining to age of pregnant mother, the highest number of age group was found between 25 and 29 years and followed by the age group between 20 and 24 years of age. The mean age of respondents was 25.68 ± SD (4.324) (minimum 20 and maximum 36). Only 24 of all pregnant mothers had hypertension induced by pregnancy and none of them had history of exposure to alcohol drinking and smoking. Almost all of pregnant mothers had single and most of them gave birth on an emmergency basis (see Table 2).

### 3.2. Fetal and Newborn Condition

Among 354 of newborns delivered by pregnant mothers, 58.14% were male. Majority of newborns had a normal birth weight and they were delivered at term. About 61.63% of all newborn were delivered when the skin incision to delivery time was greater than three minutes. Out of 46 newborns delivered under general anesthesia, 74% were delivered when induction to deliver time is more than six minutes (see Table 3).

### 3.3. Apgar Score Difference at One and Five Minutes

In this study, the effect of different factors over Apgar score of newborn babies was determined. About 30.2% of newborn baby had Apgar score below seven while about 69.8% had high Apgar score at a minute. On the other hand, about 12.8% of all newborns had low Apgar score at five minutes while about 91.2% had high Apgar score at five minutes. Generally, it was found that number of the newborn babies who had low Apgar score at one minute was reduced by more than a half as compared to Apgar score at five minutes (Table 4).

### 3.4. Determinants of Apgar Score at One Minute

Among all determinants of Apgar score at one minute age group, gestational age in terms, maternal heart rate, and type of cesarian section, maintenance agents, body mass index, and gestational type were not associated on bivariate analysis at *p* value less than 0.2, therefore excluded from multivariate analysis. It was observed from a data of multivariate analysis that skin incision to delivery time and type of anesthesia were strongly associated with a low Apgar score at *p* value less than 0.05. The odd of developing the low Apgar score when skin incision to delivery time was greater than three minutes was about three times as high as the odd of developing low Apgar score when skin incision to delivery time was less than three minutes at one minute (AOR 3.645) (95% CI (0.116-26.421)) (Table 5).

### 3.5. Determinants of Apgar Score at Five Minutes

At five minutes, induction agents, anesthesia type, body mass index, type of C/S, and age group were not associated with bivariate analysis at *p* value less than 0.2, so that it was excluded from multivariate analysis. It was observed from a data of multivariate analysis that skin incision to delivery time, gestational age, and blood pressure were strongly associated with a low Apgar score at *p* value less than 0.05. Being multiple gestations were about three times more likely to develop low Apgar score when compared to single gestation at five minutes (AOR 3.477) (95% CI (.033-16.94)) (see Table 6).

## 4. Discussion

Majority of the pregnant mothers who gave birth by cesarean section were found between age group 25 and 29 of whom the mean age was 25.28 ± SD (4.267) (minimum 20 and maximum 38). The magnitude of low Apgar score was 30.2% and 12.8% at one minute and five minutes, respectively, which is almost closest to review conducted in Uganda teaching hospital 28% [15]. In contrast to this study, the magnitude of low Apgar score in Gondar University Hospital was high (37.5%). The higher magnitude of low Apgar score in Gondar University Hospital could be attributed to preanesthetic condition as 92% of the cases were emergency C/S, fetal distress being the leading indication [16].

The result of this study also revealed that majority of newborn babies had better Apgar score both at one and five minutes. In addition, newborn who delivered under spinal anesthesia had a better Apgar score which is consistent with other similar studies [17, 18]. This is in contrast with the finding of another study which reported as there is no statistically significant difference in Apgar scores between the two groups at 1 minute. However, more neonates of the general anesthesia group appeared depressed soon after birth, needing free flow of oxygen and bag and mask ventilation [19]. The low Apgar score at one minute may be due to the result of laryngeal spasm induced by aspiration of liquor or blood during intrauterine manipulation and pregnant mothers who received general anesthesia have relatively high level of circulating catecholamine causing a reduction in uteroplacental blood flow.

In line with other study [20], low birth weight (birth weight < 2.5 kg) was found to be associated with low Apgar score at one minute and uterine incision to delivery time had association with Apgar score both at one and five minutes of postdelivery [21]. The low Apgar score in low birth weight infant may be due to very immaturity of organ and nervous systems that makes infant unable to adapt to the new environment.

Many workers have recommended that delivery is best completed 6-8 minutes after induction of general anesthesia as inhalational agents could cause neonatal depression [22, 23]. Another study reported that long induction to delivery time more than 8 minutes and uterine incision-to-delivery time more than 180 seconds has been associated with fetal hypoxia and acidosis [23].

## 5. Conclusion and Recommendation

The result of the study indicated that newborn baby who delivered under general anesthesia has relatively low Apgar score when compared to those who delivered under spinal anesthesia.

Skin incisions to delivery time and type of anesthesia were strongly associated with a low Apgar score at one minute while kin incision to delivery time, gestational age, and blood pressure were found to be associated with low Apgar score at five minutes.

Therefore, it is very important to reduce factors eliciting low Apgar score.

## Figures and Tables

**Table 1:** 

Apgar score	0	1	2
Heart rate	Absent	>100	>100
Respiratory effort	Absent	Irregular	Good
Reflex irritability	No response	Reflex irritability	Cough/sneeze
Appearance (color)	Blue or pale	Body pink with blue extremities	Completely pink
Muscle tone	Flaccid	Good tone	Spontaneous flexion

**Table 2:** Sociodemographic and personal charateristics of pregnant mothers of newborns delivered by cesarean sections under general and spinal anesthesia from January to March 2016 at Gandhi Memorial Hospital.

Variables	Category	Frequency	Percentage
Age (in years)	20-24	116	33.72
	25-29	132	38.37
	30-34	54	15.70
	35-39	42	12.21

Pregnancy state	Gravida I	128	37.20
	Gravida II	168	48.84
	Gravida III	44	12.80
	Missing	4	1.16

Types of gestation	Single	324	94.2
	Multiple gestation	20	5.8

Maternal medical condition	Hypertension	24	6.97
	Gestational diabetes mellitus	4	1.16
	Normal	316	91.87

Type of C/S	Emergency	250	72.67
	Elective	94	24.33

Type of anesthesia	Spinal	298	86.82
	General	46	13.38

**Table 3:** The fetal and newborn condition of pregnant mothers of newborns delivered by cesarean sections from January to March 2016 at Gandhi Memorial Hospital.

Variables	Category	Frequency	Percentage
Sex of the newborn baby	Male	210	58.14
	Female	144	41.86

Birth weight	1.5 to 2.5 kg	96	27.12
	2.5 to 4 kg	248	70.06
	Greater than 4 kg	10	2.82

Gestational age in terms	Preterm for GA	18	5.08
	Term for GA	310	87.57
	Postterm for GA	26	7.35

Skin incision to delivery time	≤3 minutes	132	38.37
	>3minutes	212	61.63

**Table 4:** Apgar score difference at one and five minutes among newborns delivered by cesarean sections from January to March 2016 at Gandhi Memorial Hospital.

Variable	Category	At one minute		At five minutes	
		Frequency	Percentage	Frequency	Percentage
Apgar score	Less than seven	104	30.2	44	12.8
	Seven to ten	240	69.8	300	91.2

**Table 5:** Factors affecting Apgar score at one minute among newborns delivered by cesarean sections from January to March 2016 at Gandhi Memorial Hospital.

Variable	Category		Sig.	COR	AOR
	High score	Low score		95% C.I.	95% C.I.
Type of anesthesia					
General	24	22	.021	2.244 (1.375-10.183)	3.668 (.276-10.145)
Spinal	216	82	∗	∗	∗
Skin incision to delivery time					
<3 minutes	114	18	∗	∗	∗
>3 minutes	128	84	.032	3.225 (.035-6.218)	3.645 (0.116-26.421)
Birth weight					
1.5-2.5 kg	24	72	.112	2.182 (0.024-17.415)	3.418 (2.126-12.099)
2.5-4 kg	216	32	∗	∗	∗
Blood pressure					
Normotension	226	94	∗	∗	∗
Hypertension	14	10	.456	.223 (.004-46.839)	1.229 (.012-37.277)

**Table 6:** Factors affecting Apgar score at five minutes among newsborns delivered by cesarean sections under general and spinal anesthesia from January to March 2016 at Gandhi Memorial Hospital.

Variable	Category		Sig.	COR	AOR
	High score	Low score		95% C.I.	95% C.I.
Gestational age					
Term	292	18	∗	∗	∗
Postterm	12	14	0.02	.037 (.4612-5.483)	3.824 (.024-26.94)
Preterm	6	12	0.03	2.17 (.272-10.332)	5.477 (.313-38.12)
Uterine incision to delivery time					
<3 minutes	118	14	∗	∗	∗
>3 minutes	182	30	.012	.126 (.025-10.482)	4.65 (2.134-16.463)
Birth weight					
1.5-2.5 kg	80	16	.215	1.122 (.574-23.135)	3.156 (.026-18.352)
2.5-4 kg	220	28	∗	∗	∗
Blood pressure					
Normotension	290	32	∗	∗	∗
Hypertension	12	12	.034	.187 (.277-11.453)	5.289 (.147-28.35)
Gestational type					
Single	288	36	∗	∗	∗
Multiple	12	8	.236	1.23 (.012-4.45)	1.477 (0.024-12.84).

## Data Availability

The data used in this study was collected by data collectors and submitted to authors, who are willing to share the data upon request from peer researchers.

## Abbreviations

CS: Cesarian section
ICD: International Classification of Diseases.
